# Establishment of a Human Gastric Cancer Xenograft Model in Immunocompetent Mice Using the Microcarrier-6

**DOI:** 10.1155/2020/1893434

**Published:** 2020-04-04

**Authors:** Yanzhen Bi, Quanyi Wang, Yonghong Yang, Quanquan Wang, Kai Zhang, Xiaobei Zhang, William C. Cho, Zhenfeng Shu, Jiannan Li, Lili Liu, Chuanping Si, Feng Hong

**Affiliations:** ^1^Difficult and Complicated Liver Diseases and Artificial Liver Center, Beijing Youan Hospital, Capital Medical University, Beijing, China; ^2^Department of Pathology, Affiliated Hospital of Jining Medical University, Jining, China; ^3^Institute of Liver Diseases, Affiliated Hospital of Jining Medical University, Jining, China; ^4^Department of Neuromuscular Disease, The Third Hospital of Hebei Medical University, Shijiazhuang, China; ^5^Department of General Surgery, The Second Hospital of Jilin University, Changchun, China; ^6^Department of Clinical Oncology, Queen Elizabeth Hospital, Hong Kong, China; ^7^Shanghai Meifeng Biotechnology Co., Ltd, Shanghai, China; ^8^Department of Anorectal Surgery, Affiliated Hospital of Jining Medical University, Jining, China; ^9^Institute of Immunology and Molecular Medicine, Jining Medical University, Jining, China

## Abstract

Gastric cancer is among the most common malignant tumors of the digestive tract. Establishing a robust and reliable animal model is the foundation for studying the pathogenesis of cancer. The present study established a mouse model of gastric carcinoma by inoculating immunocompetent mice with MKN45 cells using microcarrier. Sixty male C57BL/6 mice were randomly divided into three groups: a 2D group, an empty carrier group, and a 3D group, according to the coculture system of MKN45 and the microcarrier. The mouse models were established by hypodermic injection. Time to develop tumor, rate of tumor formation, and pathological features were observed in each group. In the 3D group, the tumorigenesis time was short, while the rate of tumor formation was high (75%). There was no detectable tumor formation in either the 2D or the empty carrier group. Both H&E and immunohistochemical staining of the tumor xenograft showed characteristic evidence of human gastric neoplasms. The present study successfully established a human gastric carcinoma model in immunocompetent mice, which provides a novel and valuable animal model for the cancer research and development of anticancer drugs.

## 1. Introduction

With approximately one million newly diagnosed cases annually, gastric cancer poses a serious threat to the health and lives of people worldwide [1, 2]. However, the pathogenesis and mechanisms of metastasis of gastric cancer have yet to be completely clarified. *In vivo* models of gastric cancer are essential tools for exploration of the biological characteristics of this cancer and potential novel treatment options [3, 4]. Human-derived gastric cancer xenograft models have been established in immunodeficient mice, such as nude mice and mice with severe combined immunodeficiency. However, immunodeficient mice are not easily reared and are costly. Importantly, immunodeficient mice do not reflect the important roles of the immune system in tumor development and progression. Thus, the establishment of a novel human gastric cancer xenograft model in mice with a normal immune system is of great significance [5, 6]. In the present study, MKN45, human gastric cancer cells and the microcarrier were cocultured, and immunocompetent mice were subsequently inoculated with the suspension, successfully establishing a novel human-derived gastric cancer xenograft.

## 2. Materials and Methods

### 2.1. Materials

Roswell Park Memorial Institute- (RPMI-) 1640 culture medium, trypsin, fetal bovine serum, and penicillin were purchased from Gibco (Thermo Fisher Scientific, Waltham, MA, USA). Rabbit anti-human carbohydrate antigen 199 (CA199), cytokeratin 7 (CK-7), and caudal type homeobox 2 (CDX-2) monoclonal antibodies were purchased from Abcam (Cambridge, UK). The microcarrier-6 was purchased from Elyon Biotechnologies LLC (Gaithersburg, MD, USA).

### 2.2. Experimental Animals

Sixty 6–8-week-old male C57BL/6 mice weighing 22–25 g were purchased from Jinan Pengyue Experimental Animal Breeding Co., Ltd. (license number: SCXK 20140007, Shandong Province, China) and housed in a specific pathogen-free animal center at the Affiliated Hospital of Jining Medical College (Shandong Province, China). All animal experiments were performed with the approval of the Institutional Animal Care and Use Committee of the Affiliated Hospital of Jining Medical University and carried out in accordance with the approved guidelines.

### 2.3. Cell Lines

The MKN45 human gastric cancer cell line was purchased from Cell Bank of the Chinese Academy of Science (Shanghai, China) and cultured in RPMI-1640 medium containing 10% fetal bovine serum and 100 U/mL penicillin at 37°C with 5% CO_2_. The culture medium was changed every 24 h, and the cells harvested by digestion with 0.25% trypsin.

### 2.4. Establishment of a Three-Dimensional (3D) Tumor Cell Culture Model

**Figure 1:**
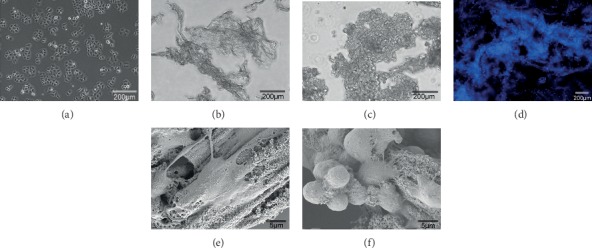
3D coculture system of MKN45 cells under the microscope and the electron microscope. (a) In the 2D culture environment, the MKN45 cells exhibited an irregular polygon shape. (b) The microcarrier-6 type C; irregular “maze”-like structure. (c) Following a 24 h coculture, MKN45 cells adhered well to the microcarrier-6, as observed by microscopy. Irregular cell clusters were observed surrounding the MKN45 cells adhered to the microcarrier-6. (d) Following a 24 h coculture, DAPI staining showed a large number of MKN45 cells adhered to the microcarrier-6 scaffold. (e) Multilayer porous structure of microcarriers can be observed under the scanning electron microscope. (f) MKN45 cells adhered to the microcarriers, and the cells adhered to the surface formed cell clusters.

The microcarrier-6 was soaked in 75% ethanol for 24 h, washed three times with 1x phosphate-buffered saline, and incubated in RPMI-1640 medium for 24 h. Subsequently, the microcarrier-6 was modified via a 3 h incubation with stromal cell-derived factor-1 alpha (SDF-1*α*) and vascular endothelial growth factor (VEGF), both at a concentration of 100 ng/mL. Cells in the logarithmic growth phase were counted following trypan blue staining and adjusted to a concentration of 2 × 10^7^/mL when the viability was >95%. The MKN45 cell suspension was mixed with the modified microcarrier-6 and incubated at 37°C with 5% CO_2_ for a further 24 h to saturate the cells with the microcarrier, as observed by microscopy (Figures 1(c) and 1(d)).

### 2.5. Animal Grouping

The 60 C57BL/6 mice were randomly divided into three groups (20 mice per group): the two-dimensional (2D) group (animals inoculated with 1 × PBS containing 2 × 10^6^ MKN45 cells), the empty carrier group (animals inoculated with 1 × PBS containing 30 *μ*g microcarrier-6), and the 3D group (animals inoculated with 1 × PBS containing 2 × 10^6^ MKN45 cells and 30 *μ*g microcarrier-6).

### 2.6. Animal Model Generation

The 3D tumor cell culture model was established on the first day of the experiment and incubated for 24 h. On the second day, the cultured cell-microcarrier complexes were washed three times in 1 × PBS and gently mixed with 1 × PBS to adjust the concentration to 2 × 10^7^ cells and 300 *μ*g microcarrier-6 per 1 mL of suspension, and placed on ice for later use. MKN45 cells in the logarithmic growth phase were harvested, trypsinized, washed three times with 1 × PBS, and resuspended in 1 × PBS at a concentration of 2 × 10^7^ cells/mL, which was placed on ice for later use. The modified microcarrier-6 was washed three times with 1 × PBS, resuspended in 1 × PBS at a concentration of 300 *μ*g/mL, and placed on ice for later use. Each mouse in all three experimental groups was inoculated with 100 *μ*L of the corresponding solution under the right axillary crest.

### 2.7. Indicator Detection and Pathological Examination

Following inoculation, the mice were housed separately by group, and daily appetite, activities, and weight were monitored. The time for local tumor formation and tumor volume in each mouse was recorded. Once tumors were visible, the long diameter (a) and short diameter (b) of each tumor were measured daily, and the tumor volume was calculated according to the tumor volume formula [7]: V = ½ × a × b^2^. Tumor-bearing mice were sacrificed in three batches: 10, 20, and 30 days postinoculation. Tumor tissues were completely removed from each mouse, and tumor quality, texture, and degree of infiltration and necrosis were recorded. Tumor tissues were subsequently fixed in 4% neutral buffered formaldehyde and stained with hematoxylin and eosin (H&E). The EnVision two-step staining technique was used for immunohistochemistry. The staining results were determined based on the positive granular cytoplasmic staining of the tumor. Negative (−ve) staining was defined as <5% positively stained cells, and positive (+ve) staining was defined as ≥5% positively stained cells.

### 2.8. Statistical Analysis

The SPSS 13.0 software (IBM SPSS, Chicago, IL, USA) was used for statistical analysis. Measurement data are presented as means ± standard error of the mean (SEM). The differences between the mean values of each group were compared using one-way analysis of variance (ANOVA) or the Kruskal−Wallis test as appropriate. *P* < 0.05 is considered a statistically significant difference.

## 3. Results

### 3.1. Establishment of the In Vitro MKN45 3D Tumor Culture System Using the Microcarrier-6

**Figure 2:**
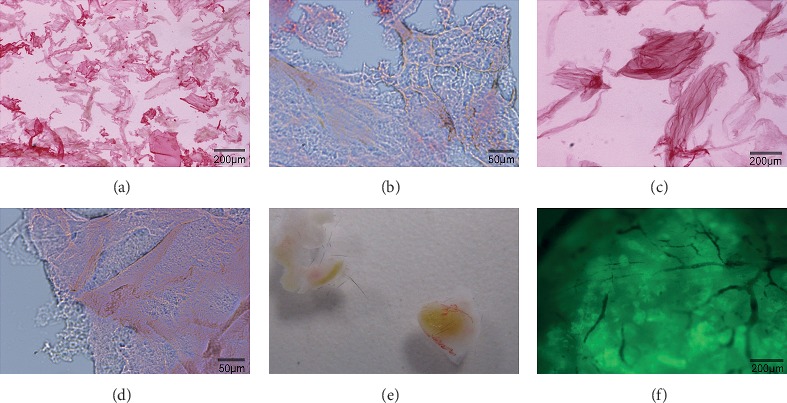
The characteristics of the microcarrier-6 by microscopy. (a, b) Type C microcarrier-6, small volume, large aperture. (c, d) Type M microcarrier-6, large volume, small aperture. The pore size, the positive surface charge density, and the size of the microcarrier-6 can be adjusted by chemical synthesis. The microcarrier-6 is multilayered and cross-linked to form an irregular “maze”-like structure with sufficient space (b, d). The microcarrier-6 is directly embedded in the skin of C57BL/6 mice for 5 weeks, inducing blood vessel ingrowth (e, f). (e) Visually, the red surfaces are blood vessels. (f) Under the microscope, the dendritic shadows are blood vessels.

The microcarrier-6 is a novel microcarrier composed of positively electrifiable organic polymers with a multilayered porous structure. The pore size, the positive surface charge density, and the size of the microcarrier-6 can be adjusted by chemical synthesis. Type C microcarrier-6, small volume, large pore size, and many times of space folding, is mainly used for 3D cell culture and drug sensitivity experiments (Figures 2(a) and 2(b)). Type M microcarrier-6, large volume, small pore size, and low internal folding times, is mainly used for tissue engineering research (Figures 2(c) and 2(d)). Type C microcarrier-6 was used in our study. The microcarrier-6 is a pure organic compound that is not prone to contamination, has no impurities, is low in immunogenicity and metabolism, and is highly biocompatible, providing a stable microenvironment for cell growth (Figures 2(a)–2(d)). In addition, the microcarrier-6 is directly embedded in the skin of mice, inducing blood vessel ingrowth (Figures 2(e) and 2(f)), which can provide sufficient blood supply for tumor cell growth. MKN45 cells adhered rapidly in the 2D environment, and the cell morphology observed by microscopy following 24 h of culture exhibited an irregular polygon shape (Figure 1(a)). The microcarrier-6 exhibited an irregular or long spindle shape and a loose texture (Figure 1(b)). Following a 24 h coculture of MKN45 and the modified microcarrier-6, MKN45 cells adhered well to the microcarrier and reached confluence. In addition, irregular cell clusters were observed surrounding the MKN45 cells adhered to the microcarrier-6 (Figure 1(c)). The MKN45 cell-microcarrier complexes were stained with 4′,6-diamidino-2-phenylindole (DAPI) solution, revealing a large number of MKN45 cells adhered to the microcarrier-6 scaffold (Figure 1(d)). Multilayer porous structure of microcarriers can be observed under scanning electron microscope (Figure 1(e)). MKN45 cells adhered to the microcarriers, and the cells adhered to the surface formed cell clusters (Figure 1(f)).

### 3.2. Mouse Survival and Tumor Formation

**Figure 3:**
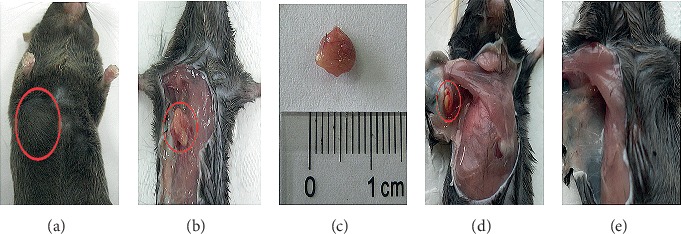
Histological changes in the injection sites of the different groups. (a, b, c) Large subcutaneous masses were observed 10 days following inoculation in the 3D group. (b, c) The xenograft tissues were easily separated from the adjacent tissues and presented as relatively regular shapes, mostly round or oval, and grayish white or grayish red in color. (d) Small subcutaneous masses were observed with a yellow color in the empty carrier group. (e) No subcutaneous mass was observed in the 2D group.

No significant change in appetite, coat, or body weight was noted among the groups during the experiment (Table 1). Mice in the 3D group showed a slight decrease in activity one week postinoculation. No animals died in any group, and no tumor formation was found in the empty carrier or 2D groups during the 30-day experiment. Subcutaneous masses in 15 of the 20 mice in the 3D group were identified via palpation 7–10 days following inoculation, suggesting a tumor formation rate of 75% (Table 1). Tumor xenografts grew rapidly and were observed as subcutaneous masses approximately 10 days following inoculation (Figure 3(a), Supplementary [Supplementary-material supplementary-material-1]). Tumor xenografts grew to 0.5–1.0 cm^3^ in size from 2 to 3 weeks postinoculation. The tumor xenograft tissues were easily separated from the adjacent tissues and presented as relatively regular shapes, mostly round or oval, grayish white or grayish red in color, and with an abundant peripheral blood supply (Figures 3(b) and 3(c) and Supplementary [Supplementary-material supplementary-material-1]). There were histological changes of the injection site in the empty carrier group, but no changes in the 2D group (Figures 3(d) and 3(e)). Small subcutaneous masses were observed with a yellow color in the empty carrier group (Figure 3(d)).

### 3.3. H&E Staining

**Figure 4:**
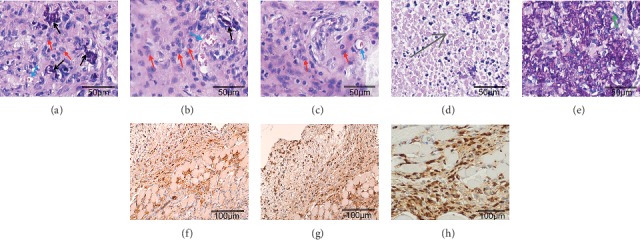
H&E and immunohistochemical staining of a human gastric cancer xenograft (200x). (a) A large number of heterotypic cells, residual microcarriers, and abundant blood vessels were observed, 10 days postinoculation. (b, d) A small number of residual microcarriers (b) and a large number of necrotic lesions (d), 20 days postinoculation. (c) Almost no residual microcarriers can be found, 30 days postinoculation. (e) A large number of microcarriers and a small number of inflammatory cells were seen in the empty carrier group. Blue arrows refer to blood vessels, red arrows indicate heterotypic cells, and black arrows show residual microcarriers (a, b, c). Gray arrows refer to necrotic lesions (d), and green arrows refer to inflammatory cells (e). The human gastric cancer xenograft displayed diffuse and strong immunoreactivity in the cytoplasm or nuclei of the cancer cells. (f) CA199, (g) CK-7, and (h) CDX-2.

Histomorphology, as seen by light microscopy, showed a large number of disordered and atypical cells in the tumor xenografts from mice in the 3D group (Figures 4(a)–4(c)). A large number of heterotypic cells, residual microcarriers, and abundant blood vessels were observed, 10 days postinoculation (Figure 4(a)). A small number of residual microcarriers (Figure 4(b)) and a large number of necrotic lesions (Figure 4(d)) were observed, 20 days postinoculation. However, the microcarriers were substantially eliminated 30 days postinoculation (Figure 4(c)). A large number of microcarriers and a small number of inflammatory cells were observed in the empty carrier group, but no tumor cells were found (Figure 4(e)).

### 3.4. Immunohistochemistry

Immunohistochemistry was carried out in animals euthanized 20 days following tumor xenograft. The tumor xenograft tissues showed positive CA199, CK-7, and CDX-2 staining (Figures 4(f)–4(h)), further confirming that the atypical cells were human-derived tumor cells.

## 4. Discussion

Animal models have been extensively used to study the pathogenesis and metastatic mechanisms of gastric cancer and play an extremely important role in the evaluation of the efficacy and toxicity of therapeutic drugs [8–10]. At present, gastric cancer animal models mainly include the following: the induction model, the transplantation model, and the genetic engineering model [11]. Among the three types of models, the transplantation model is easily operated and thus widely used. The type of experimental animals, tumor xenografts, and the site and route of transplantation have been considered factors that affect the success of xenograft models [11]. Mice with defects in immune function, such as nude mice and mice with severe combined immunodeficiency (SCID), are commonly used for xenograft models [11, 12]. However, due to the comparatively expensive cost, short life span, and the lack of immune response to tumors in immunodeficiency mice, we selected immunocompetent C57BL/6 mice for the xenograft model to avoid the shortcomings of immunodeficiency mice. It is generally believed that the occurrence of gastric cancer is not related to the level of estrogen, and the incidence of gastric cancer in males is higher than that in females. Thus, we used male mice as models. In addition, the present study used MKN45 human gastric cancer cells to establish the *in vitro* model mainly because of the strong invasive and metastatic characteristics of the cell line [13]. Moreover, the underarm subcutaneous region and ectopic transplantation were selected as the site and route of tumor cell transplantation, respectively, as a result of the abundant blood supply and loose tissue structure of this area, which facilitates tumor growth in mice [14].

As a bridge between the 2D cell culture system and the animal model, 3D cell culture system better simulates the microenvironment of tumor cell growth and has become an attractive topic in current research [15–17]. 3D culture of cancer cells will form tumor organoids, and mouse tumor xenograft models based on tumor organoids have begun to be applied to the screening of anticancer drugs [18, 19]. The present study used the novel microcarrier-6 and MKN45 cells for the coculture experiments to successfully establish a 3D growth model. We directly embedded the microcarrier-6 into the subcutaneous tissue of mice to allow blood vessels to enter the microcarriers, presenting a specific advantage that is not achieved with other microcarriers. Studies have reported mild immunosuppression of immunocompetent mice to achieve resistance to human tumor cells, successfully establishing a tumor-bearing mouse model [20]; however, to date, there are no reports of successful ectopic transplantation of a human tumor in normal mice under nonimmunosuppressive conditions. In addition, when we adjusted the concentration of MKN45 cells to 2 × 10^8^ cells/mL and immediately injected 100 *μ*L MKN45 cell suspension subcutaneously to each mouse, stable tumor formation was not achieved due to the strong immune clearance of the ectopically transplanted human tumor cells. The microcarrier-6 used in the present study exhibited low immunogenicity and a loose texture with numerous pores in the center, facilitating tumor cell growth. The microcarrier-6 also acted as a barrier to block the immune cells from directly killing the tumor cells. The modified microcarrier-6 allowed blood vessels to easily grow inside the tumor, providing suitable conditions for the rapid growth of tumor cells.

Cellular immunity is the main army against tumor growth; the cells involved mainly include T cells, natural killer cells, macrophages, and dendritic cells [6, 12]. Due to the irregular “maze”-like structure of the microcarrier-6, it can act as a short-term barrier and block the direct killing of tumor cells by immune cells to some extent. In addition, three hours prior to the experiment, the microcarrier-6 was further modified with SDF-1*α* and VEGF to accelerate blood vessel formation and provide a blood supply for rapid tumor growth, which surpassed the antitumor immune effect of the mice. Simultaneously, we found that macrophages in peripheral blood and splenic tissues of the mice were significantly decreased during the early stage of transplanted tumor formation, as compared with the normal control group (Supplementary [Supplementary-material supplementary-material-1]). The number of bone marrow macrophages gradually decreased between the empty carrier group, the 2D group, and the 3D group, but there was no statistical significance (Supplementary [Supplementary-material supplementary-material-1]). Compared with the empty carrier group, the number of spleen T lymphocytes in the 2D group was reduced, but there was no statistical significance (Supplementary [Supplementary-material supplementary-material-1]). The number of spleen T lymphocytes in the 3D group was significantly less than that in the 2D group (Supplementary [Supplementary-material supplementary-material-1]). This suggests that the growth of the tumor inhibited the immune system, allowing the tumor to grow rapidly.

The present study established a MKN45 cell 3D growth model, successfully establishing a human gastric cancer xenograft model in immunocompetent mice in the 3D group, whereas the mice in the 2D and empty carrier groups did not form any tumors. This xenograft model was characterized by the rapid growth of tumors, which could be palpated by hand 7–10 days postinoculation and reached optimal growth by 10–15 days postinoculation; the tumor volume was 0.5–1.0 cm^3^ approximately 20 days following inoculation. A large number of cells with nuclear atypia that had infiltrated muscle and fat tissues were detected by H&E staining. Residual microcarrier-6 was surrounded by inflammatory cells and formed granulomas with a large area of necrosis in the center, which was likely caused by an insufficient blood supply due to rapid tumor growth. These phenomena are consistent with tumor development in humans. Moreover, the abundant capillaries were mainly located in the periphery of the tumors. At present, there is no specific tumor marker for gastric cancer; however, CA199, CK-7, and CDX-2 are commonly used as markers of gastrointestinal tumors [21–23]. Thus, we chose these three markers for the labeling and identification of human-derived gastric cancer cells. Immunohistochemical staining of CA199, CK-7, and CDX-2 was positive, further confirming that heteromorphic cells were human gastric cancer cells.

## 5. Conclusions

We successfully established a human gastric cancer xenograft model in immunocompetent mice. This model can reflect the interaction between the body's immune system and tumors and has broad application prospects in the future research on tumor immunity as well as new drug research and development [24].

## Figures and Tables

**Table 1:** Body weight and tumor volume of mice at different time points.

Time (day)	Empty carrier group		2D group		3D group	
	Body weight (g)	Volume (mm^3^)	Body weight (g)	Volume (mm^3^)	Body weight (g)	Volume (mm^3^)
1	24.15 ± 1.01	0	24.53 ± 1.06	0	24.44 ± 1.23	0
3	24.32 ± 1.21	0	24.71 ± 1.11	0	24.56 ± 1.39	0
5	24.87 ± 1.12	0	24.74 ± 1.27	0	24.73 ± 1.23	7.36 ± 3.15 (*n* = 8)
7	25.12 ± 1.37	0	24.91 ± 1.43	0	24.87 ± 0.85	35.18 ± 6.24 (*n* = 13)
10	25.67 ± 1.51	0	25.34 ± 1.27	0	25.13 ± 1.31	95.76 ± 10.27 (*n* = 15)
14	25.93 ± 0.96	0	25.96 ± 1.08	0	25.56 ± 1.19	415.35 ± 30.71 (*n* = 15)
21	26.65 ± 1.02	0	26.37 ± 1.07	0	26.14 ± 1.28	534.27 ± 45.18 (*n* = 15)
30	27.31 ± 1.35	0	26.85 ± 1.37	0	26.37 ± 1.05	527.16 ± 50.62 (*n* = 15)

Data are shown as the mean ± SEM.

## Data Availability

The data used to support the findings of this study are available from the corresponding author upon request.

## Supplementary Materials

Supplementary Materials The changes of inflammatory cells and other tumorigenic data. Supplementary Figure 1: the changes of inflammatory cells in tumor-bearing mice during the early stage of transplanted tumor formation. Supplementary Figure 2: the changes of inflammatory cells in different groups during the early stage of transplanted tumor formation. Supplementary Figure 3: tumor-bearing mice and transplanted tumor tissues.

## References

[1] Bray F., Ferlay J., Soerjomataram I., Siegel R. L., Torre L. A., Jemal A. (2018). Global cancer statistics 2018: GLOBOCAN estimates of incidence and mortality worldwide for 36 cancers in 185 countries. *CA: A Cancer Journal for Clinicians*.

[2] Global Burden of Disease Cancer Collaboration, Fitzmaurice C., Allen C. (2017). Global, regional, and national cancer incidence, mortality, years of life lost, years lived with disability, and disability-adjusted life-years for 32 cancer groups, 1990 to 2015: a systematic analysis for the global burden of disease study. *JAMA Oncology*.

[3] Kuwata T., Yanagihara K., Iino Y. (2019). Establishment of Novel Gastric Cancer Patient-Derived Xenografts and Cell Lines: Pathological Comparison between Primary Tumor, Patient-Derived, and Cell-Line Derived Xenografts. *Cells*.

[4] Tellez-Gabriel M., Cochonneau D., Cadé M., Jubelin C., Heymann M. F., Heymann D. (2019). Circulating tumor cell-derived pre-clinical models for personalized medicine. *Cancers*.

[5] Basel M. T., Narayanan S., Ganta C. (2018). Developing a xenograft human tumor model in immunocompetent mice. *Cancer Letters*.

[6] Wang M., Busuttil R. A., Pattison S., Neeson P. J., Boussioutas A. (2016). Immunological battlefield in gastric cancer and role of immunotherapies. *World Journal of Gastroenterology*.

[7] Ogawa K., Mukai T., Asano D. (2007). Therapeutic effects of a 186Re-complex-conjugated bisphosphonate for the palliation of metastatic bone pain in an animal model. *Journal of nuclear medicine : official publication, Society of Nuclear Medicine*.

[8] Ding L., El Zaatari M., Merchant J. L. (2016). Recapitulating human gastric cancer pathogenesis: experimental models of gastric cancer. *Advances in experimental medicine and biology*.

[9] Liu X., Meltzer S. J. (2017). Gastric cancer in the era of precision medicine. *Cellular and Molecular Gastroenterology and Hepatology*.

[10] Song Y., Tong C., Wang Y. (2018). Effective and persistent antitumor activity of HER2-directed CAR-T cells against gastric cancer cells in vitro and xenotransplanted tumors in vivo. *Protein & Cell*.

[11] Poh A. R., O'Donoghue R. J., Ernst M., Putoczki T. L. (2016). Mouse models for gastric cancer: matching models to biological questions. *Journal of Gastroenterology and Hepatology*.

[12] Zitvogel L., Pitt J. M., Daillère R., Smyth M. J., Kroemer G. (2016). Mouse models in oncoimmunology. *Nature Reviews Cancer*.

[13] Busuttil R. A., Liu D. S., di Costanzo N., Schröder J., Mitchell C., Boussioutas A. (2018). An orthotopic mouse model of gastric cancer invasion and metastasis. *Scientific Reports*.

[14] Zheng M. J., Wang J., Chen Y. W. (2012). A novel mouse model of gastric cancer with human gastric microenvironment. *Cancer Letters*.

[15] Jaeger S., Duran-Frigola M., Aloy P. (2015). Drug sensitivity in cancer cell lines is not tissue-specific. *Molecular Cancer*.

[16] Mitra A., Mishra L., Li S. (2013). Technologies for deriving primary tumor cells for use in personalized cancer therapy. *Trends in Biotechnology*.

[17] Rimann M., Graf-Hausner U. (2012). Synthetic 3D multicellular systems for drug development. *Current Opinion in Biotechnology*.

[18] Vlachogiannis G., Hedayat S., Vatsiou A. (2018). Patient-derived organoids model treatment response of metastatic gastrointestinal cancers. *Science*.

[19] Weeber F., Ooft S. N., Dijkstra K. K., Voest E. E. (2017). Tumor organoids as a pre-clinical cancer model for drug discovery. *Cell Chemical Biology*.

[20] Bennett J. A., Pilon V. A., MacDowell R. (1985). Evaluation of growth and histology of human tumor xenografts implanted under the renal capsule of immunocompetent and immunodeficient mice. *Cancer Research*.

[21] Heatley M. K. (2008). Immunohistochemical biomarkers of value in distinguishing primary ovarian carcinoma from gastric carcinoma: a systematic review with statistical meta-analysis. *Histopathology*.

[22] Hui L., Rixv L., Xiuying Z. (2015). A system for tumor heterogeneity evaluation and diagnosis based on tumor markers measured routinely in the laboratory. *Clinical Biochemistry*.

[23] Kim H. S., Lee J. S., Freund J. N. (2006). CDX-2 homeobox gene expression in human gastric carcinoma and precursor lesions. *Journal of Gastroenterology and Hepatology*.

[24] Choi R. S., Lai W. Y. X., Lee L. T. C. (2019). Current and future molecular diagnostics of gastric cancer. *Expert Review of Molecular Diagnostics*.

