# Supplementary material for: Establishment of a Human Gastric Cancer Xenograft Model in Immunocompetent Mice Using the Microcarrier-6
Source: Biomed Res Int. 2020 Apr 4;2020:1893434. doi: 10.1155/2020/1893434 (PMC7165317; doi:10.1155/2020/1893434)
Supplement: Supplementary Materials — The changes of inflammatory cells and other tumorigenic data. Supplementary Figure 1: the changes of inflammatory cells in tumor-bearing mice during the early stage of transplanted tumor formation. Supplementary Figure 2: the changes of inflammatory cells in different groups during the early stage of transplanted tumor formation. Supplementary Figure 3: tumor-bearing mice and transplanted tumor tissues. [file 1893434.f1.doc]

**Establishment of a Human Gastric Cancer Xenograft Model in Immunocompetent Mice Using the Microcarrier-6**

Yanzhen Bi^1#^, Quanyi Wang^2#^, Yonghong Yang^3#^, Quanquan Wang^4^, Kai Zhang^5^, Xiaobei Zhang^3^, William C. Cho^6^, Zhenfeng Shu^7^, Jiannan Li^5^, Lili Liu^8^, Chuanping Si^9*^ and Feng Hong^3*^

**Supplementary materials and methods**

**Animal and Treatment**

In the present study, we performed two additionalgroups of animal studies, which are normal group (n=10) and 3D group (n=10) to observe the changes of inflammatory cells in tumor-bearing mice during the early stage of transplanted tumor formation (Supplementary Figure 1). The normal mice group did not receive any treatment, while 3D group were treated as before (2.6. Animal Model Generation). On the tenth day, the mice were sacrificed, bone marrow and spleen tissues were collected for examination, and check whether the tumor was formed. 3D (+) represented tumor-bearing mice in 3D group, while 3D (-) represented non-tumor mice in 3D group in Supplementary Figure 1.

In addition, 24 8-week-old male C57BL/6 mice were used for experiments to observe the changes of inflammatory cells between group 2D, group empty-carrier and group 3D during the early stage of transplanted tumor formation (Supplementary Figure 2). Mice were treated as before (2.6. Animal Model Generation) in different groups (n=8). On the tenth day, The mice were sacrificed, bone marrow and spleen tissues were collected for examination.

**Flow cytometry**

For phenotypic staining, cells were washed twice with PBS containing 1% FBS and 0.1% NaN_3_, subsequently incubated with mouse primary antibodies against CD3, CD11b, F4-80, CD86 and CD206 for 30 min at 4°C according to the manufacture’s instruction. After twice washings with PBS, cells were analyzed using a FACSCalibur flow cytometer (BD Biosciences, New Jersey, USA). An isotype control was used for each antibody.

**Supplementary Figures**


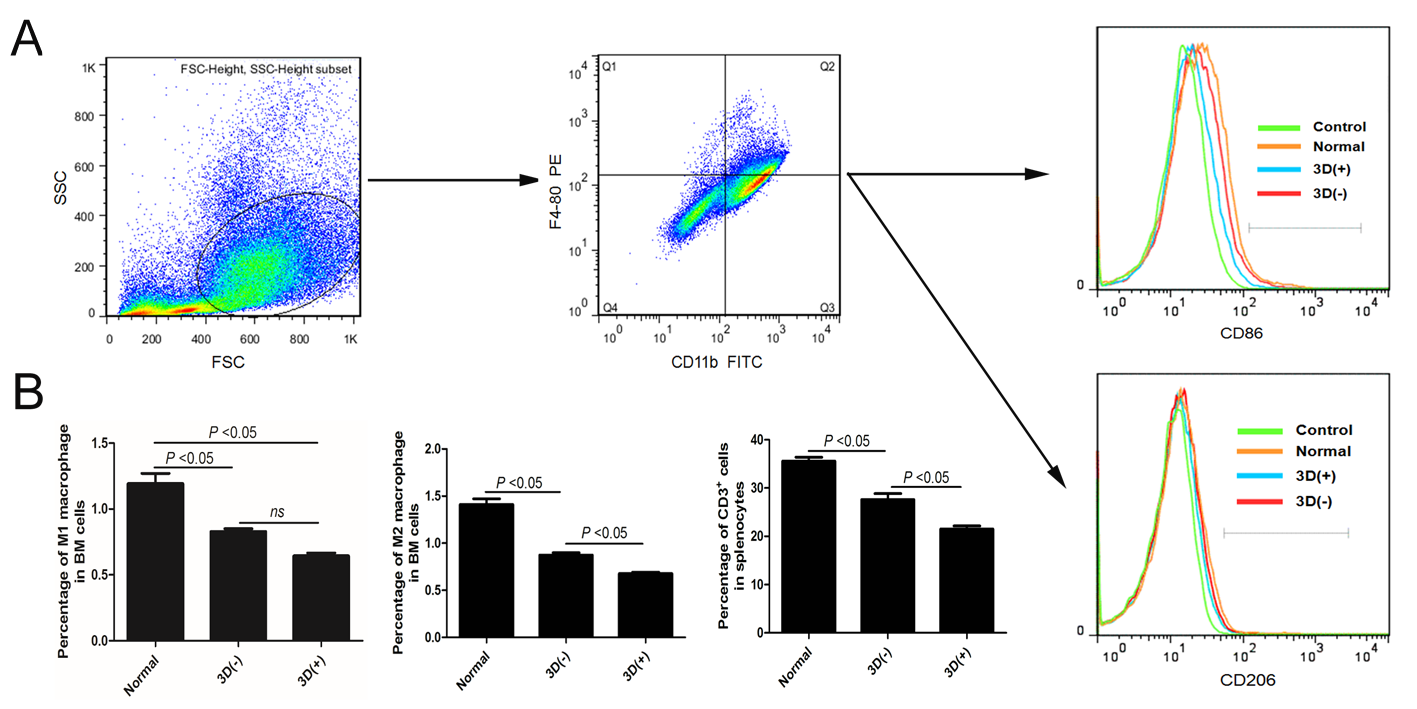


**Supplementary Figure 1** The changes of inflammatory cells in tumor-bearing mice during the early stage of transplanted tumor formation. The number of bone marrow macrophages (A, B) and spleen T lymphocytes (B) in tumor-bearing mice were significantly decreased, as compared with normal mice. Data are shown as the mean ± SEM, as determined by ANOVA; ns denotes *P* > 0.05.


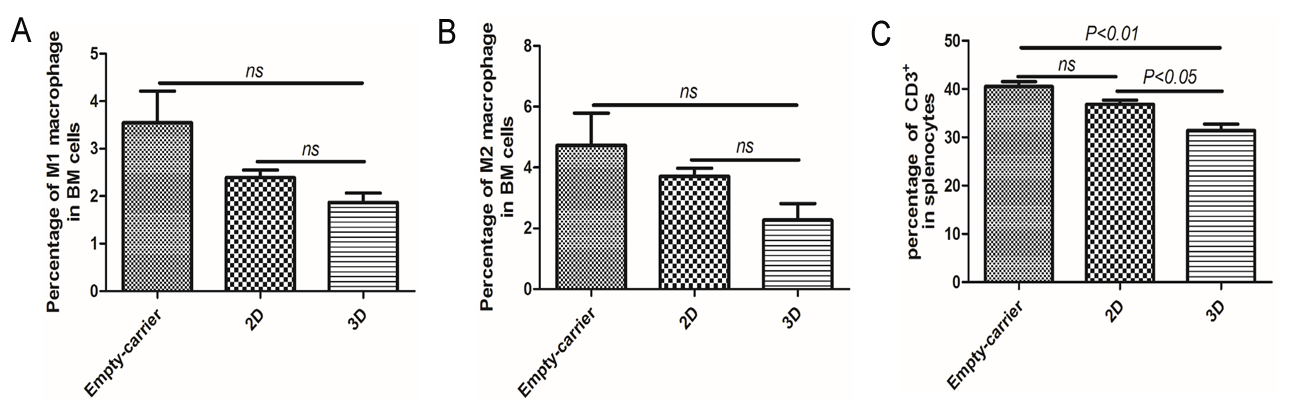


**Supplementary Figure 2** The changes of inflammatory cells in different groups during the early stage of transplanted tumor formation. The number of bone marrow Macrophages gradually decreased between the empty-carrier group, the 2D group, and the 3D group, but there was no statistical significance (A, B). Compared with the empty-carrier group, the number of spleen T lymphocytes in the 2D group was reduced, but there was no statistical significance (C). The number of spleen T lymphocytes in the 3D group was significantly less than that in the 2D group (C). Data are shown as the mean ± SEM, as determined by ANOVA; ns denotes *P* > 0.05.


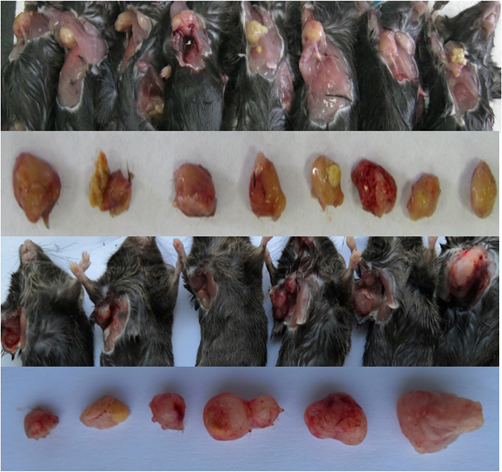


**Supplementary Figure 3** Tumor-bearing mice and transplanted tumor tissues.
